# NURSES' EXPERIENCES CARING FOR PERSONS LIVING WITH DEMENTIA AND CHRONIC LEG WOUNDS IN THE COMMUNITY

**DOI:** 10.1093/geroni/igac059.1621

**Published:** 2022-12-20

**Authors:** Justine Sefcik, Olivia Hernandez, Isabella Stoll, Zachary Hathaway, Ellen Bass, Rose Ann DiMaria-Ghalili

**Affiliations:** Drexel University, Philadelphia, Pennsylvania, United States; Drexel University, Philadelphia, Pennsylvania, United States; Drexel University, Philadelphia, Pennsylvania, United States; Drexel University, Philadelphia, Pennsylvania, United States; Drexel University, Philadelphia, Pennsylvania, United States; Drexel University, College of Nursing and Health Professions, Philadelphia, Pennsylvania, United States

## Abstract

A gap exists with understanding the care required for community-dwelling persons living with dementia (PLWD) who have chronic wounds. As one step to address this gap we explored the experiences of nurses who care for community-dwelling PLWD that have chronic diabetic foot and venous leg ulcers. A qualitative descriptive approach with a conventional content analysis was undertaken. We conducted 5 focus groups with Home Health Nurses (n = 13) and nurses holding specialty certifications (e.g., wound care) (n = 3); 87.5% female, 69% White, non-Hispanic, mean age = 52 (range 32-67), mean years of experience as a nurse = 23.8. The main themes identified were 1) the challenges with dementia (e.g., confusion, taking dressings off), 2) concerns about the person (e.g., medical issues, financial considerations), and 3) adjusting interventions (for wounds and behavioral symptoms). Study findings can inform the develop of future novel interventions for PLWD and chronic wounds.

